# The E3 Ubiquitin-Ligase Bmi1/Ring1A Controls the Proteasomal Degradation of Top2α Cleavage Complex – A Potentially New Drug Target

**DOI:** 10.1371/journal.pone.0008104

**Published:** 2009-12-01

**Authors:** Iris Alchanati, Carmit Teicher, Galit Cohen, Vivian Shemesh, Haim M. Barr, Philippe Nakache, Danny Ben-Avraham, Anna Idelevich, Itzchak Angel, Nurit Livnah, Shmuel Tuvia, Yuval Reiss, Daniel Taglicht, Omri Erez

**Affiliations:** Proteologics Ltd, Rehovot, Israel; University of Minnesota, United States of America

## Abstract

**Background:**

The topoisomerases Top1, Top2α and Top2β are important molecular targets for antitumor drugs, which specifically poison Top1 or Top2 isomers. While it was previously demonstrated that poisoned Top1 and Top2β are subject to proteasomal degradation, this phenomena was not demonstrated for Top2α.

**Methodology/Principal Findings:**

We show here that Top2α is subject to drug induced proteasomal degradation as well, although at a lower rate than Top2β. Using an siRNA screen we identified Bmi1 and Ring1A as subunits of an E3 ubiquitin ligase involved in this process. We show that silencing of Bmi1 inhibits drug-induced Top2α degradation, increases the persistence of Top2α-DNA cleavage complex, and increases Top2 drug efficacy. The Bmi1/Ring1A ligase ubiquitinates Top2α *in-vitro* and cellular overexpression of Bmi1 increases drug induced Top2α ubiquitination. A small-molecular weight compound, identified in a screen for inhibitors of Bmi1/Ring1A ubiquitination activity, also prevents Top2α ubiquitination and drug-induced Top2α degradation. This ubiquitination inhibitor increases the efficacy of topoisomerase 2 poisons in a synergistic manner.

**Conclusions/Significance:**

The discovery that poisoned Top2α is undergoing proteasomal degradation combined with the involvement of Bmi1/Ring1A, allowed us to identify a small molecule that inhibits the degradation process. The Bmi1/Ring1A inhibitor sensitizes cells to Top2 drugs, suggesting that this type of drug combination will have a beneficial therapeutic outcome. As Bmi1 is also a known oncogene, elevated in numerous types of cancer, the identified Bmi1/Ring1A ubiquitin ligase inhibitors can also be potentially used to directly target the oncogenic properties of Bmi1.

## Introduction

Anticancer drugs targeting topoisomerases (Top) are some of the most widely used chemotherapeutic agents. These drugs are type specific; they target either Top1 or Top2α and Top2β. The Top2 poisons (*e.g.* Etoposide, Teniposide (VM26) and Doxorubicin) increase the steady state levels of an intermediate state of the reaction, producing a Top2-DNA cleavage complex comprised of Top2 covalently bound to a double strand DNA break [1]. Eventually the Top2-DNA cleavage complex forms cytotoxic DNA lesions that trigger cell cycle arrest and cell death. Top2 poisons convert the enzyme into a DNA damaging agent with a stochiometric relationship, one DNA double strand break for every drug molecule bound to a Top2 enzyme. Thus sensitivity to Top2 poisons is dependent on high levels of Top2-DNA cleavage complexes. Moreover, the efficacy of Top2-targeted agents reflects the persistence of drug-induced cleavage complexes in cells [2].

Proteasomal degradation of Top2 is one of the mechanisms that decrease the persistence of drug-Top2-DNA complex thus contributing to the emergence of drug resistance and reduced efficacy. While Top2β was shown to be specifically degraded following treatment with Top2 drugs [3], [4], physiological conditions, such as glucose deprivation and hypoxia, have been shown to induce degradation of Top2α [5] leading to decreased Top2α levels, rendering cells resistant to Top2-targeted drugs such as etoposide and doxorubicin [6]. Hence, inhibition of ubiquitin-dependent degradation of topoisomerases may improve long-term therapeutic efficacy of topoisomerase-targeted drugs. Further support for a degradation based resistance mechanism is obtained from the fact that proteasome inhibition circumvents solid tumor resistance to Top2-directed drugs [7]. Inhibition of the E3 ubiquitin ligase that directs the drug-Top-DNA complex for degradation should stabilize the cleavage complex in a similar manner and concomitantly increase drug-induced efficacy. Inhibiting a specific E3 ligase is expected to be superior to inhibiting the proteasome as it is expected to have much lower side effects.

Here we demonstrate first that Top2α, similar to Top2β, is degraded following a treatment with the Top2 drug teniposide (VM26) although at a slower rate then Top2β. We describe the identification of Bmi1 and Ring1A as subunits of an E3 ubiquitin ligase complex that is involved in both, drug-induced Top2α degradation and low-glucose induced Top2α degradation. Silencing of either Bmi1 or Ring1A by RNAi reduces drug-induced Top2α degradation and correlates with increased drug efficacy in various cell-lines, while overexpression of Bmi1 induces increased ubiquitination of Top2α. A purified complex formed by Bmi1 and Ring1A is shown to ubiquitinate immunopurified Top2α. We describe a high-throughput assay for the discovery of small-molecule inhibitors of Bmi1/Ring1A. A compound discovered using this assay prevents degradation of Top2α induced by a Top2 drug and increases the efficacy of Top2 drugs in a synergistic manner.

## Materials and Methods

### Reagents and Antibodies

All cell-lines were purchased from the American Type Culture Collection (ATCC). Dicer substrate 27-nucleotide long siRNA duplexes were purchased from IDT Integrated DNA technologies (Coralville, IA). The sequences of the siRNA used are specified in Table S1. All siRNA transfection were conducted using Saint-Red siRNA transfection reagent (Synvolux Therapeutics, Groningen, Holland) and all plasmid transfection were conducted using Lipofectamine2000 reagent (Invitrogen, Carlsbad, CA). Teniposide (VM26) was purchased from Alexis Biochemicals. Reagents for homogenous time resolved FRET (HTRF^®^) were purchased from Cisbio Bioassays (Bagnols-sur-Cèze, France). For generation of antibodies against Bmi1, a GST fusion protein containing residues 228–326 of Bmi1 was constructed by PCR amplification. The plasmid was expressed in *E. coli BL21*, purified by glutathione chromatography, the GST removed by PreScission™ (GE Healthcare, Life Sciences) digest and sera produced in rabbits (Sigma-Aldrich, Israel). Antibodies for Ring1A, Top1, Top2α and Top2β were purchased from Santa Cruz Biotechnology (Santa Cruz, CA). Antibody for Ring1B was purchased from MBL International (Woburn, MA). Chemical libraries were purchased from IBS (Moscow, Russia), Chemdiv (San-Diego, CA) and Timtec (Newark, Delaware).

### Expression Plasmids

Human Bmi1 and Ring1A were PCR amplified from I.M.A.G.E clones 4138748 and 6142438 respectively [I.M.A.G.E. Consortium, http://image.llnl.gov [8], obtained from Geneservice Ltd, UK]. Bmi1 was cloned into pcDNA3.1/V5-HisA (Invitrogen) to include a C-terminal V5-HIS tag that was later modified to include a C-terminal FLAG tag. Full length Top2α was generated by combining two IMAGE clones (4101949 and 6501467) into pCMV-SPORT6. HA-tagged ubiquitin was a gift from Prof. Yosef Yarden, Weizmann Inst. of Science.

### Recombinant Proteins

Ubiquitin activating enzyme E1 was expressed in Sf9 cells and purified as previously described [9]. Various ubiquitin conjugating enzymes and HA-ubiquitin, Biotin-ubiquitin and FLAG-ubiquitin were purchased from Boston Biochem (Cambridge, MA). For production of Bmi1 and Ring1A, codon optimized cDNAs (DNA2.0) were cloned into pGEX-6P (GE Healthcare, Life Sciences) in frame with Glutathione S-transferase (GST). The Bmi1 fusion protein also contained an N-terminal V5 tag and a C-terminal six-histidine tag. The Ring1A fusion protein also contained an N-terminal HA tag and a C-terminal six-histidine tag. For expression in *E. coli BL21*, proteins were induced with 0.2 mM IPTG and lysates were purified by sequential glutathione (GE Healthcare, Life Sciences) and Ni-NTA (Qiagen, Valencia, CA) chromatography.

### Cell Viability Assay

Cells were plated at low confluence (about 10% for testing compounds and 40% for siRNA assays) in 96-well plates. Twenty-four hours later the cells were transfected with 100 nM siRNA or treated with compounds and incubated for 72 hr. When testing the effect of siRNA, thirty-two hours post-transfection the cells were treated for 16 hours with DMSO or VM26 and then for additional twenty-four hours with fresh medium. Viability was measured using Cell Proliferation Reagent WST-1 (Roche, Mannheim, Germany). LD50 was calculated using Prism software (GraphPad software, CA).

### VM26-Induced Top2α Degradation Assay

HeLa cells, either, two days post transfection with 100 nM siRNA, or following one hour pre-treatment with the tested compound, were treated with 100 µM VM26 for the indicated time at 37°C. Where indicated, at the end of the incubation the medium was replaced with fresh medium without drugs for 30 minutes to facilitate the recovery of ubiquitin and ubiquitin-like non-conjugated Top2 from the DNA. Harvested cells were resuspended in alkaline lysis buffer and treated with S7 nuclease (Roche) as previously described [3]. The extracts were separated by SDS-PAGE (7.5%) followed by Western-blot with Top2α antibody.

### Top2α Cleavable Complex Detection

HeLa cells, two days after siRNA transfection, were treated with VM26 (100 µM) with or without MG132 (50 µM) for 0, 0.5 or 6 hr. DNA bound proteins were separated as previously described [10]. Samples of 1 µg DNA-protein complex were spotted on to a PVDF membrane using a Dot-Blot apparatus and detected with anti Top2α antibody.

### In-Vivo Top2α Ubiquitination

HeLa cells transfected with plasmids encoding V5-tagged Bmi1 and Ring1A as indicated, were treated with 50 µM MG132 for 30 minutes with or without 100 µM VM26. The cells were extracted with hot lysis buffer (1% SDS, 1 mM EDTA in PBS), boiled 5 min at 95°C and sonicated to break DNA. The extracts were separated by SDS-PAGE (6.5%) followed by immuno-detection with Top2α antibodies.

### In-Vivo Bmi1 Ubiquitination Assay

HeLa cells transfected with plasmids encoding V5-tagged Bmi1, Ring1A and HA-tagged ubiquitin as indicated were treated for five hours with either solvent or compound. Cells were harvested and lysed in RIPA (50 mM Tris, pH 7.5; 150 mM NaCl; 1% NP-40, 0.5% Na-DOC, 0.1% SDS, 1 mM EDTA and a protease inhibitor cocktail). Followed by three freeze-thaw cycles, Bmi1 was immunoprecipitaed using anti-V5 antibodies and separated by SDS-PAGE (10%), followed by Western-blot with anti-HA (ubiquitin) and anti-V5 (Bmi1) antibodies.

### Gel Based Bmi1/Ring1A Ubiquitination Assay

Recombinant Bmi1, Ring1A or Bmi1/Ring1A complex produced in bacteria were used in a cell free ubiquitination reactions. Typical reaction contains 5 nM recombinant E1, 100 nM E2, 5–20 nM E3, 1 µM ubiquitin and 40 mM Tris-HCl buffer pH 7.6, 5 mM MgCl_2_, 2 mM ATP and 0.1 mM DTT. Reaction was terminated after 60 minutes incubation at 37°C with 5 mM of EDTA, separated by SDS-PAGE (10%), followed by Western-blot with anti-HA (Ring1A) and anti-V5 (Bmi1) antibodies.

### HTRF^®^ Based Bmi1/Ring1A Ubiquitination Assay

Ubiquitination reactions were as for the gel-based assay but contained Flag-tagged ubiquitin. Ubiquitin-chain elongation was quantified by HTRF^®^ (excitation at 320 nm, emission at 620 and 665) between adjacent Flag-ubiquitin molecules, using anti-Flag-cryptate and anti-Flag-XL665 antibodies (CisBio) using a dedicated fluorescence plate reader (RubyStar, BMG Labtech, Offenburg Germany). Activity is calculated as the ratio of emission of acceptor XL-665 (665 nm) to emission of the donor cryptate (620 nm) X 10,000.

### *In Vitro* Bmi1/Ring1A Mediated Top2α Ubiquitination Assay

HeLa cells were transfected with Flag-tagged Top2α. Twenty-four hours post transfection cells were harvested, extracted in lysis buffer and immuno-precipitated with anti-Flag conjugated beads (Sigma). The bead-bound proteins were used as a substrate in an ubiquitination reaction, containing: recombinant Bmi1/Ring1A as E3, UbcH5a as E2, E1, ATP and either biotin-tagged or HA-tagged ubiquitin. At the end of the reaction beads were washed, proteins eluted in SDS buffer (95°C, 5 min) and separated by SDS-PAGE (6.5%), followed by detection with either streptavidin-horseradish peroxidase (HRP) or anti-HA-HRP.

### Immunofluorescence Assay

Cells (HeLa and A375) were fixed for 15 min in 4% paraformaldehyde at ambient temperature, neutralized with 0.125 M glycine for 10 min, and then permeabilized with PBS, 0.1% Triton X-100 for 2 minutes, and washed three times with PBS. Fixed cells were blocked with 10% normal donkey serum and stained with Bmi1 sera using goat anti rabbit-cy2 as secondary antibody. Cells were visualized using a confocal microscopy (Carl Zeiss Axiovert 100 M).

## Results

### Teniposide Induces Proteasomal Degradation of Top2α

**Figure 1:**
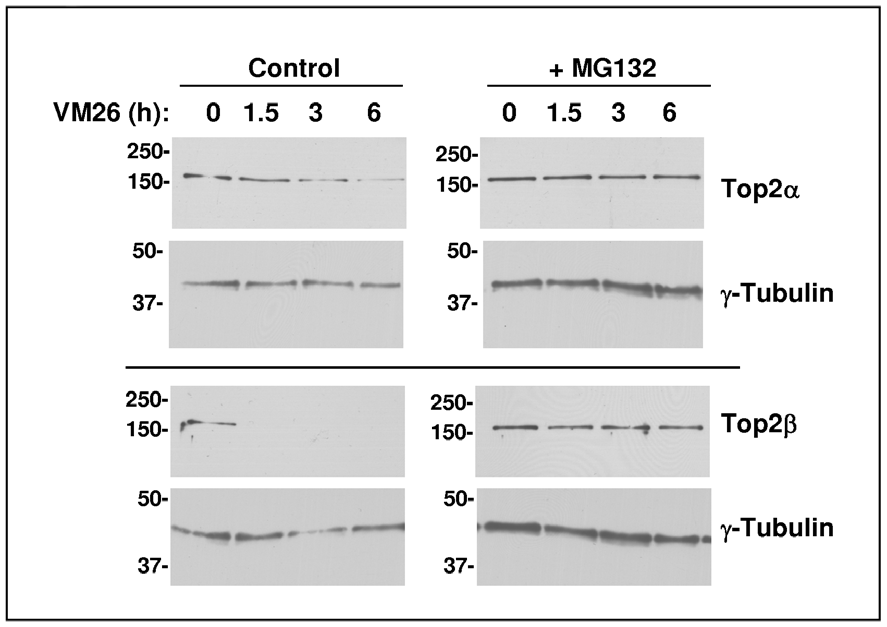
VM26 induces proteasomal degradation of Top2α. HeLa cells were treated for the indicated time with 100 µM VM26 and with control solvent or 20 µM of proteasome inhibitor MG132. At the end of the incubation the medium was replaced with a fresh medium without drugs for 30 minutes to facilitate the recovery of ubiquitin and ubiquitin-like non-conjugated Top2 from the DNA. Top2α and β proteins were recovered from the DNA by alkaline lysis and S7 nuclease treatment and their level was determined by Western-blot with Top2α or β antibodies. The level of γ-Tubulin is shown as control.

As it was reported that Top2α is proteasomally degraded under physiological conditions such as low glucose and hypoxia [6], we tested whether treatment with a Top2 drug can also lead to its proteasomal degradation. To that end we treated cells with VM26 for up to 6 h and monitored the levels of both Top2α and Top2β (Fig. 1). While Top2β levels decrease to bellow the detectable level within 1.5 h, the decrease in the levels of Top2α is much slower, with an estimated half-life of about 4 h. The degradation of both Top2 isozymes, is prevented by the addition of the proteasome inhibitor MG132, indicating that the degradation is proteasomal.

### Identification of Bmi1 and Ring1A as an E3 Ubiquitin Ligase Involved in Targeting Top2α-Drug Complex for Degradation

To identify an E3 ubiquitin ligase responsible for targeting Top2-drug complexes for degradation, we conducted an siRNA-based screen. A list of 77 candidate E3 ligase genes was drawn based on various criteria. The preliminary screen conducted in HeLa cells was based on the assumption that silencing of a critical E3 will increase the toxicity of a Top2-directed drug. The screen was carried out in the presence of a sub-toxic concentration of VM26. Cells were transfected separately with two different siRNA per gene tested. Positive candidates that increased VM26 toxicity were further tested for prevention of drug-induced Top2 (α or β isoforms) degradation. Candidate targets that were positive in both assays with at least one siRNA were further characterized using additional siRNAs for the same target gene, so as to eliminate false positive identification caused by siRNA off-target artifacts.

**Figure 2:**
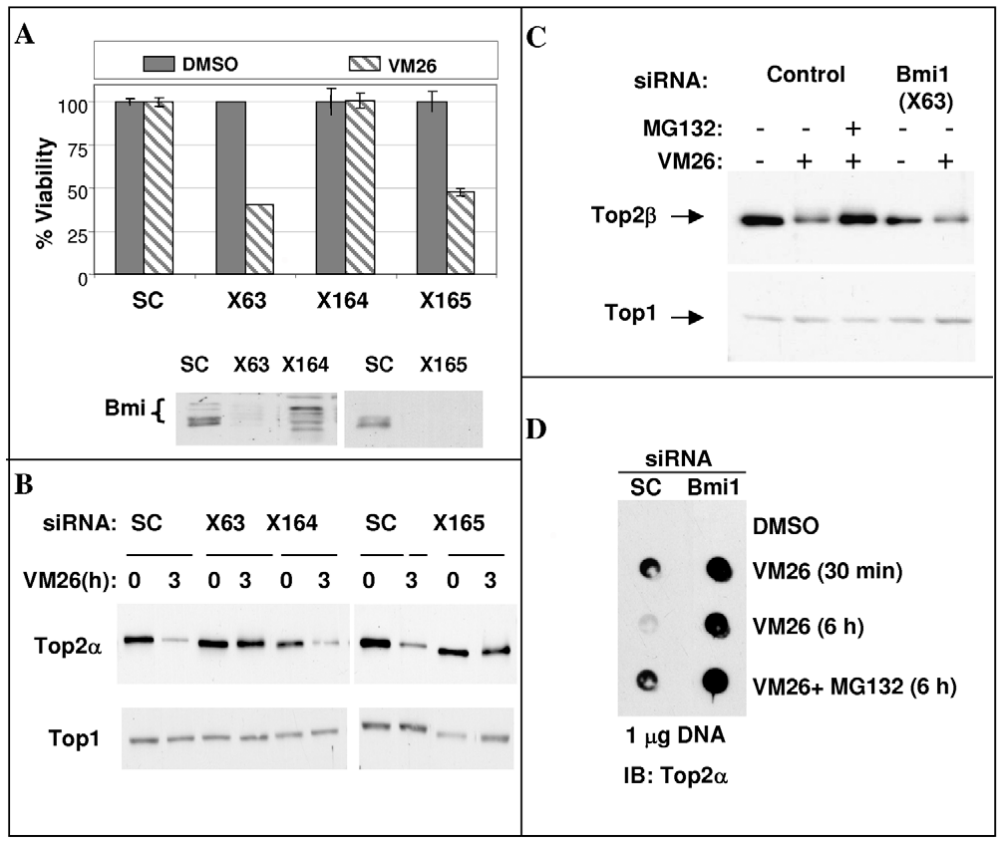
Silencing of Bmi1 increases Top2-drug-induced toxicity, inhibits drug-induced Top2α degradation and stabilizes Top2α cleavage complex. A. HeLa cells were transfected with three different siRNAs targeting Bmi1 (X63, X164, X165) and scramble siRNA (SC). Following transfection, the sensitivity to 1 µM VM26 was determined as described in the material and methods. Lower panel: Immuno-blot assay to determine the reduction of Bmi1 protein level. B. HeLa cells transfected with siRNA as described in panel A were treated 48 hours later with VM26 for the indicated time. Top2α protein was recovered from the DNA by alkaline lysis and S7 nuclease treatment and its level was determined by Western-blot with Top2α antibody. Levels of Top1 are shown as control. C. HeLa cells transfected with control or Bmi1 siRNA (X63) were treated 48 hours later with 100 µM VM26 and 25 µM MG132 for 3 hours as indicated. The level of Top2β was determined after alkaline lysis and S7 nuclease treatment by Western-blot with Top2β antibody. Levels of Top1 are shown as control. D. HeLa cells transfected with control (SC) or Bmi1 siRNA (X63) were treated 48 hours later with VM26 (100 µM) and MG132 (25 µM) as indicated. The level of Top2α cleavage complex in the cells was determined as described in the material and methods.

A single candidate, Bmi1, was scored positive in both the increased toxicity assay and the drug-induced Top2α degradation assay using two different siRNAs. Bmi1 is a RING finger protein and is a key component of the Polycomb repressive complex 1 (PRC1) that is involved in epigenetic silencing of targeted genes [11]. Three different siRNAs designed to knockdown Bmi1 expression were assayed for their effect on VM26-induced toxicity in HeLa cells (Fig. 2A). Two of the siRNAs (X63 and X165) sensitized the cells for VM26-induced toxicity, increasing the killing efficacy of VM26 by 50–60%, whereas the third (X164) had no effect. When testing the effectiveness of the three siRNAs towards reducing the expression level of Bmi1 protein, we found that X164, the only siRNA that failed to increase the efficacy of VM26, also failed to reduce expression of Bmi1 protein (Fig. 2A lower panel).

We further examined the effect of Bmi1 silencing on drug-induced degradation of the two Top2 isozymes (α or β). HeLa cells transfected with Bmi1 siRNAs were treated for the indicated time with VM26. At the end of the treatment, the total amount of Top2 proteins was determined. The results show that both siRNA that are effective at reducing Bmi1 protein level, X63 and X165, prevented the drug-induced Top2α degradation (Fig. 2B). Silencing of Bmi1 did not prevent VM26-dependent degradation of Top2β (Fig. 2C).

### Bmi1 Silencing Increases DNA-Top2α Cleavage Complex Level

Drug-induced cell toxicity is considered to be in correlation with the amount of the DNA-Top cleavage complex [2] and our hypothesis was that stabilization of this complex would lead to increased drug efficacy. We set out to check if silencing of Bmi1 also stabilizes the fraction of Top2α that is covalently bound to DNA. HeLa cells transfected with either control or Bmi1 siRNA were treated for various periods of time with either DMSO or VM26 with or without the proteasome inhibitor MG132 as indicated (Figure 2D). Genomic DNA was separated on a cesium-chloride cushion to resolve proteins covalently bound to DNA from free proteins. The pelleted genomic DNA was recovered and the amount of Top2α covalently bound to equal amounts of DNA determined by a dot-blot analysis. In DMSO treated cells Top2α was not found attached to the DNA. After 30 minutes of VM26 treatment, Top2α was found attached to DNA in both control cells and cells with reduced expression of Bmi1. In the control cells DNA-bound Top2α is eliminated after 6 hours of VM26 treatment, while this elimination is prevented by MG132, indicating it was degraded by the proteasome. In contrast, in Bmi1 knockdown cells the Top2α-DNA complex is stabilized, as it is found bound to genomic DNA also after 6 hours of drug treatment. This result strengthens the hypothesis that silencing of Bmi1 increases drug efficacy thorough stabilization of the cytotoxic Top2α-DNA cleavage complex.

### Bmi1 Silencing Increases Drug Efficacy by an Order of Magnitude

**Figure 3:**
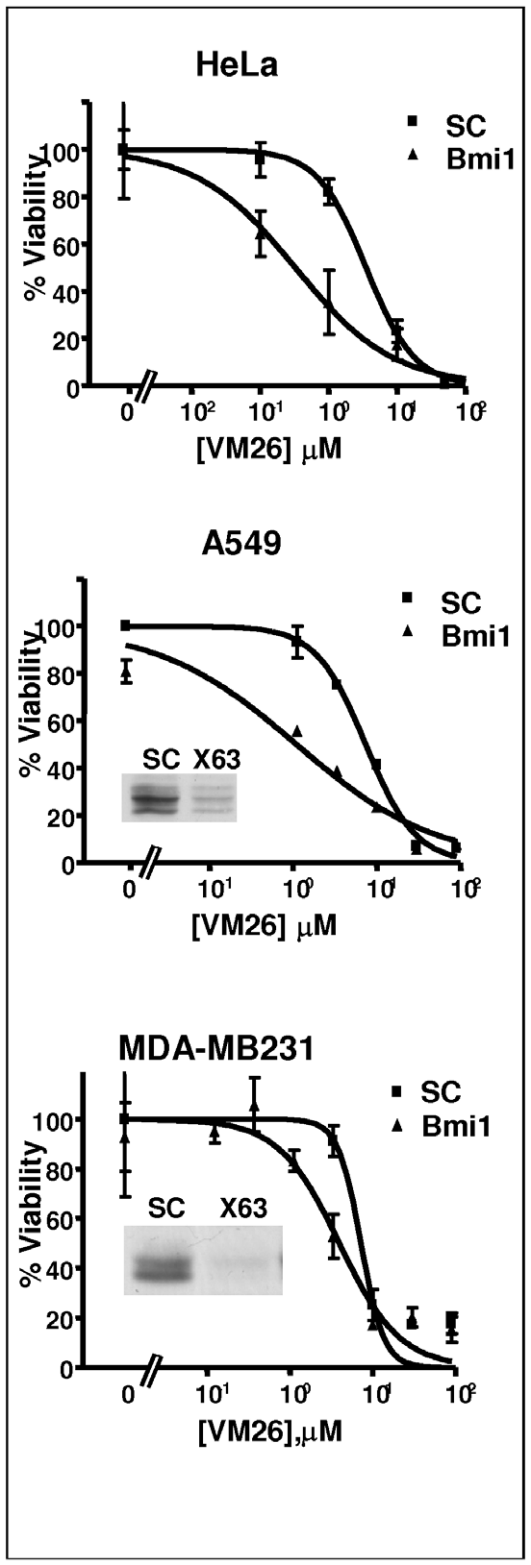
Increased toxicity of VM26 following siRNA mediated reduction of Bmi1. HeLa, A549 and MDA-MB-231 cells were transfected with Control (SC) or Bmi1 (X63) siRNAs. Following transfection, the sensitivity of the cells to different concentrations of VM26 was determined as described in material and methods. The LD50 of VM26 was calculated using Prism software (see text). The reduction of Bmi1 protein level by the siRNA is presented in the insert.

To evaluate better the degree by which Bmi1 silencing increases the sensitivity to Top2 drugs we determined the LD50 of VM26 in different cell lines transfected with Bmi1 or control siRNAs. In HeLa cervical cancer cells an LD50_VM26_ of 0.3 µM was measured with Bmi1 siRNA compared to 3.5 µM with a control siRNA, and in A549 lung cancer cells an LD50_VM26_ of 1.1 µM was measured with Bmi1 siRNA compared to 7.4 µM with the control siRNA. In both cases, Bmi1 silencing increases the efficacy of VM26 about ten-fold (Fig. 3). Similar results were obtained with HT29 colon cancer cell-lines (data not shown). The effect in MDA-MB-231 breast cancer cells was somewhat weaker, LD50_VM26_ of 3.9 µM with Bmi1 siRNA compared to 7.2 µM with control siRNA (Fig. 3). Silencing of Bmi1 in many other cells such as PC3 and DU145 prostate, MDA-MB 468 breast and RKO colon cancer cell lines had by itself a strong toxic effect (data not shown) that hindered testing sensitization to the drug. This death may be related to a pro-malignant function of Bmi1, as indeed it was reported that silencing of the Bmi1 expression promotes cancer-specific cell death [12].

### Silencing of Bmi1 Prevents Glucose Deprivation Induced Top2α Degradation

**Figure 4:**
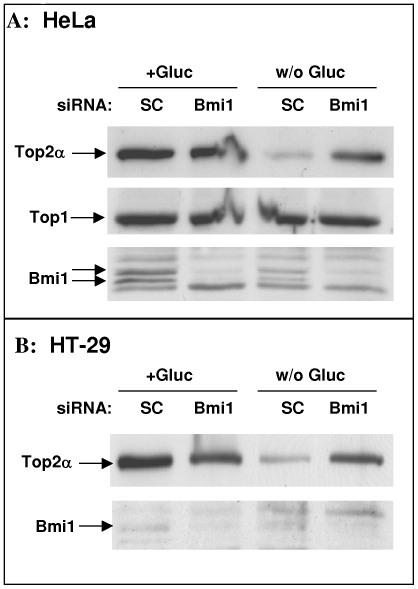
Effect of Bmi1 silencing on Top2α protein levels in glucose deprived cells. HeLa (A) and HT-29 cells (B) were transfected with either control (SC) or Bmi1 siRNA and then grown in normal medium (+Gluc) or in medium without glucose (w/o Gluc) for additional twenty-four hours. The levels of Top2α, Top1 and Bmi1 were determined by Western-blot analysis.

Previous publications show that Top2α level is reduced at physiological conditions such as glucose deprivation and hypoxia that are common in the tumor environment, and that this leads to decreased efficacy of the Top2-directed drugs [6]. The reduction in Top2α level is due to its increased proteasomal degradation, [7], [13], [14]. This led us to test whether Bmi1 silencing can prevent the low-glucose-induced Top2 degradation. Towards that end, HeLa (Fig. 4A) or HT29 (Fig. 4B) cells treated with either control or Bmi1 siRNA were grown for two days in either normal or low glucose media. In the control siRNA treated cells, the level of Top2α was reduced when cells were grown in low-glucose, while in cells transfected with Bmi1 siRNA there was no change in the levels of Top2α. These results suggest that silencing of Bmi1 has the potential to also prevent Top2-directed drug resistance emerging from reduction of Top2α level due to conditions such as glucose starvation.

### Silencing of Ring1A Prevents Drug-Induced Top2α Degradation

**Figure 5:**
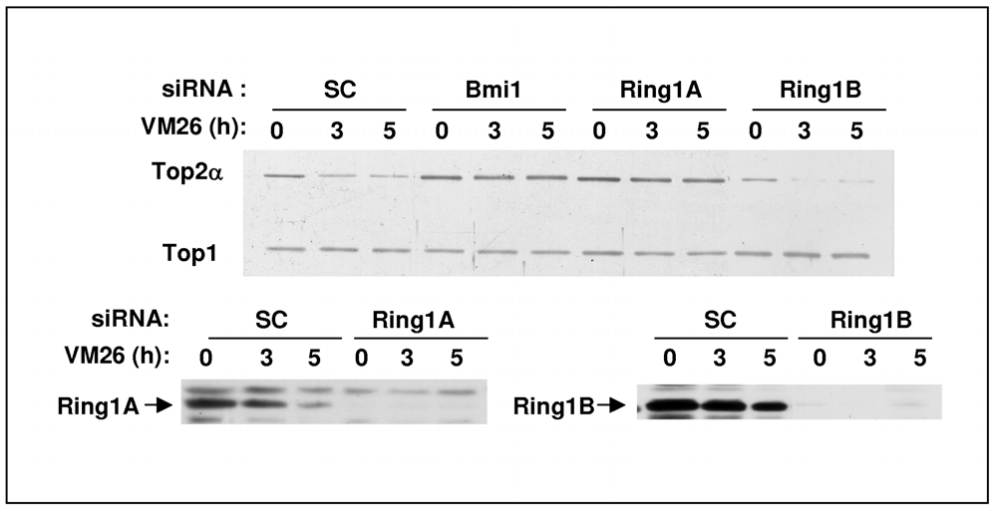
Ring1A but not Ring1B is required for VM26 induced degradation of Top2α HeLa cells transfected with siRNA targeting Bmi1, Ring1A, Ring1B or control siRNA (SC) were treated 48 hours later with 100 µM VM26 for the indicated time. Top2α protein was recovered from the DNA by alkaline lysis and S7 nuclease treatment and its level was determined by Western-blot with Top2α antibody. Levels of Top1 are shown as control. The activity of Ring1A and Ring1B targeting siRNAs was verified by Western-blot analysis with the appropriate antibodies (lower panels).

Bmi1 is a component of PRC1 [15] that is involved in Histone 2A (H2A) ubiquitination [16], [17]. PRC1 also contains the interchangeable RING finger proteins, Ring1A and Ring1B that bind to Bmi1 through their RING domains [18], [19], [20]. Therefore, to further characterize the Bmi1 E3 complex we tested the role of Ring1A and Ring1B in drug-induced Top2α degradation using an RNAi approach in HeLa cells. Verified siRNAs for Ring1A and Ring1B (Figure 5, lower panels) were tested for their effect on VM26-induced Top2α degradation (Figure 5 upper panel). The results demonstrate that while reduction of Ring1A expression prevented drug induced Top2α degradation to the same extent as a reduction of Bmi1 expression, reducing the expression of Ring1B had no effect. These results, together with the known interaction between Bmi1 and the two Ring1 proteins, suggest that Bmi1 and Ring1A form an E3-ubiquitin ligase involved in drug-induced Top2α degradation. It is interesting to note that Ring1B and not Ring1A is the catalytic subunit for PRC1 mediating ubiquitination of H2A [16] while both Bmi1 and Ring1A increase the ubiquitination efficacy of Ring1B [17].

### Bmi1 and Ring1A Form Together an Active E3 Ubiquitin-Ligase that Binds and Ubiquitinates Top2α

**Figure 6:**
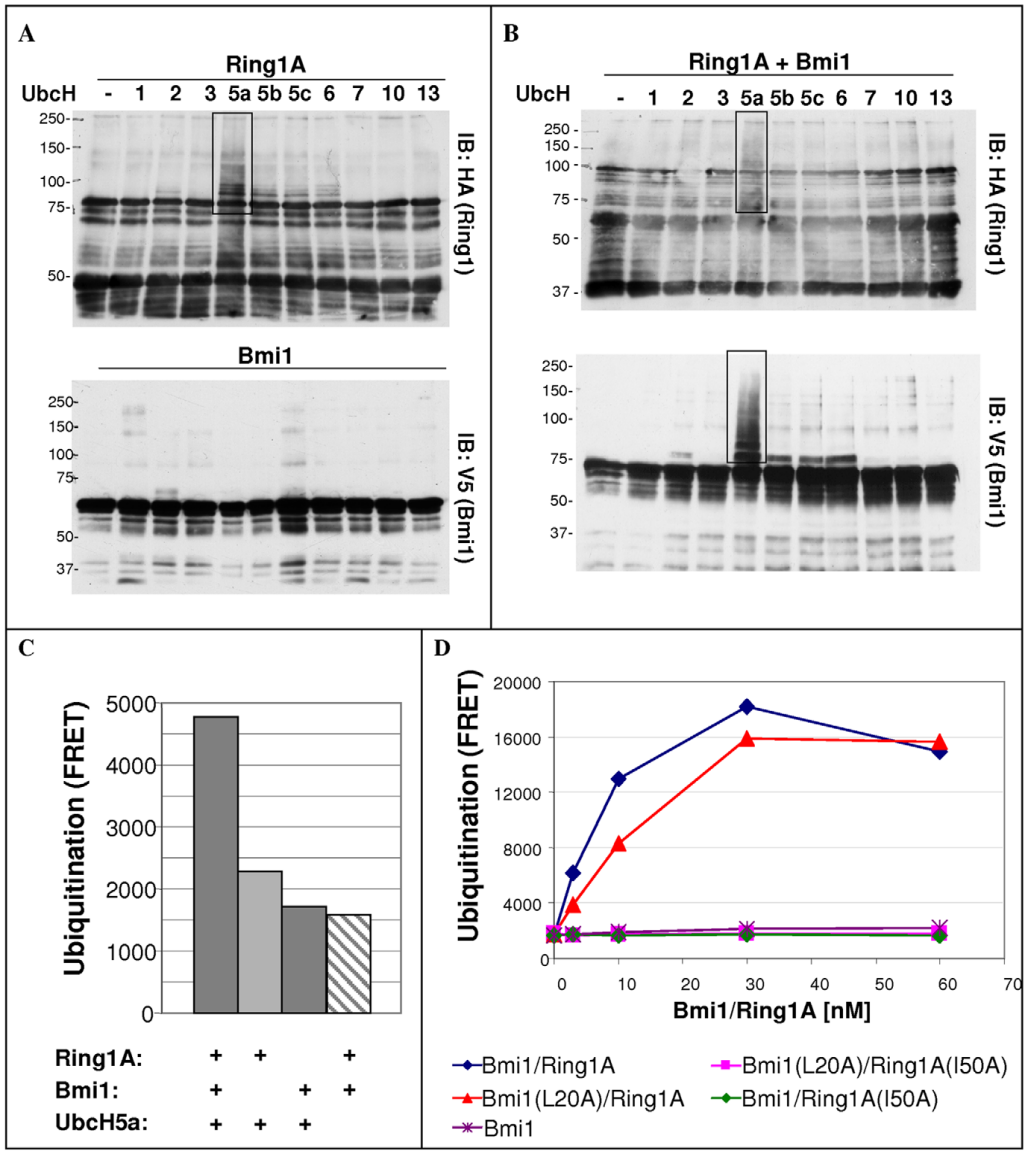
Characterization of Bmi1 and Ring1A ubiquitination activity in cell-free systems. Cell-free ubiquitination assays were carried with recombinant Bmi1 and Ring1A proteins and with different recombinant E2s. Levels of ubiquitination were determined by Western-blot analysis with indicated antibodies (A&B) or by HTRF^®^ method (C & D). A. Ubiquitination reactions with either Ring1A (top) or Bmi1 (bottom) and with different E2 enzymes. B. Ubiquitination reactions containing both Bmi1 and Ring1A, with different E2 enzymes. Western-blot was carried out for tag present on either Ring1A (top) or Bmi1 (bottom). C. Ubiquitin-chain elongation assay carried out with recombinant Bmi1 and Ring1A proteins, either separately or in combination, using UbcH5a as an E2. D. Ubiquitin-chain elongation assay carried out with either a wild-type GST-Bmi1/Ring1A purified complex or purified complexes with different combinations of RING domain mutants. A reaction containing Bmi1 only serves as a background control. The E2 enzyme utilized is UbcH5a.

Next, we wanted to characterize the ubiquitination activity of Ring1A and Bmi1. For that purpose we produced tagged recombinant Ring1A and Bmi1 proteins and tested their activity, either separately or together, in a cell-free auto-ubiquitination assay with a panel of different E2 ubiquitin-conjugating enzymes. Ubiquitination activity is assessed by monitoring the increase in molecular weight of the ubiquitinated proteins, using tag-directed antibodies. When each protein is assayed alone, Ring1A acts preferentially with UbcH5a (Fig. 6A upper panel), while Bmi1 is inactive with all the E2 enzymes tested (Fig. 6A lower panel). When Ring1A and Bmi1 are combined and assayed together, Bmi1 is highly ubiquitinated in the presence of UbcH5a (Fig. 6B lower panel) whereas ubiquitination of Ring1A is very weak (Fig. 6B upper panel). Weak Bmi1 ubiquitination is also seen with UbcH2, UbcH5b, UbcH5c and UbcH6. The preference of Bmi1/Ring1A E3 ubiquitin-ligase to UbcH5a was confirmed using the HTRF^®^-based detection method of cell-free ubiquitin-chain elongation (Fig S1). This assay measures total ubiquitination activity regardless of the ubiquitination target. The results of the HTRF^®^-based assay are in agreement with the gel-based assay showing that the Bmi1-Ring1A complex has a preference for UbcH5a as an E2. Using this type of assay we also confirmed the gel-based results that Ring1A alone has weak ubiquitination activity, Bmi1 alone is inactive while assaying Bmi1 and Ring1A together results in significant increase in the activity (Fig. 6C).

There are two possible explanations for the observed ubiquitination of Bmi1 when it is assayed together with Ring1A (Fig. 6B and 6C). One explanation is that ubiquitination of Bmi1 is mediated by Ring1A activity; while a second explanation is that binding to Ring1A activates Bmi1 to become an active E3 ubiquitin ligase. To distinguish between these two possibilities we carried the HTRF^®^-based ubiquitin-chain elongation assay with wild-type or L20A RING domain mutant of Bmi1 and either wild-type Ring1A or a I50A RING domain mutant of Ring1A. The L20A and I50A point mutations in Bmi1 and Ring1A respectively, are similar to mutations in other RING proteins where it was demonstrated to prevent binding to the E2 [21], [22], [23], but does not interfere with functional dimerization with a partner RING domain protein [24]. Strong ubiquitination activity is detected only when Bmi1, wild-type or mutant, are combined with functional Ring1A (Fig. 6D). When Bmi1 is combined with the I50A Ring1A mutant protein, the complex is inactive (Fig. 6D), indicating that binding of Ring1A is not sufficient for Bmi1 to become active. The activity of Bmi1(L20A)-Ring1A complex was comparable to the activity of wild-types Bmi1-Ring1A complex suggesting that the E2 binding site on Bmi1 is not contributing to the activity. Hence, we conclude that Bmi1 and Ring1A form an active heterodimeric E3 ubiquitin ligase, where Ring1A is the active component and Bmi1 greatly enhances its activity.

**Figure 7:**
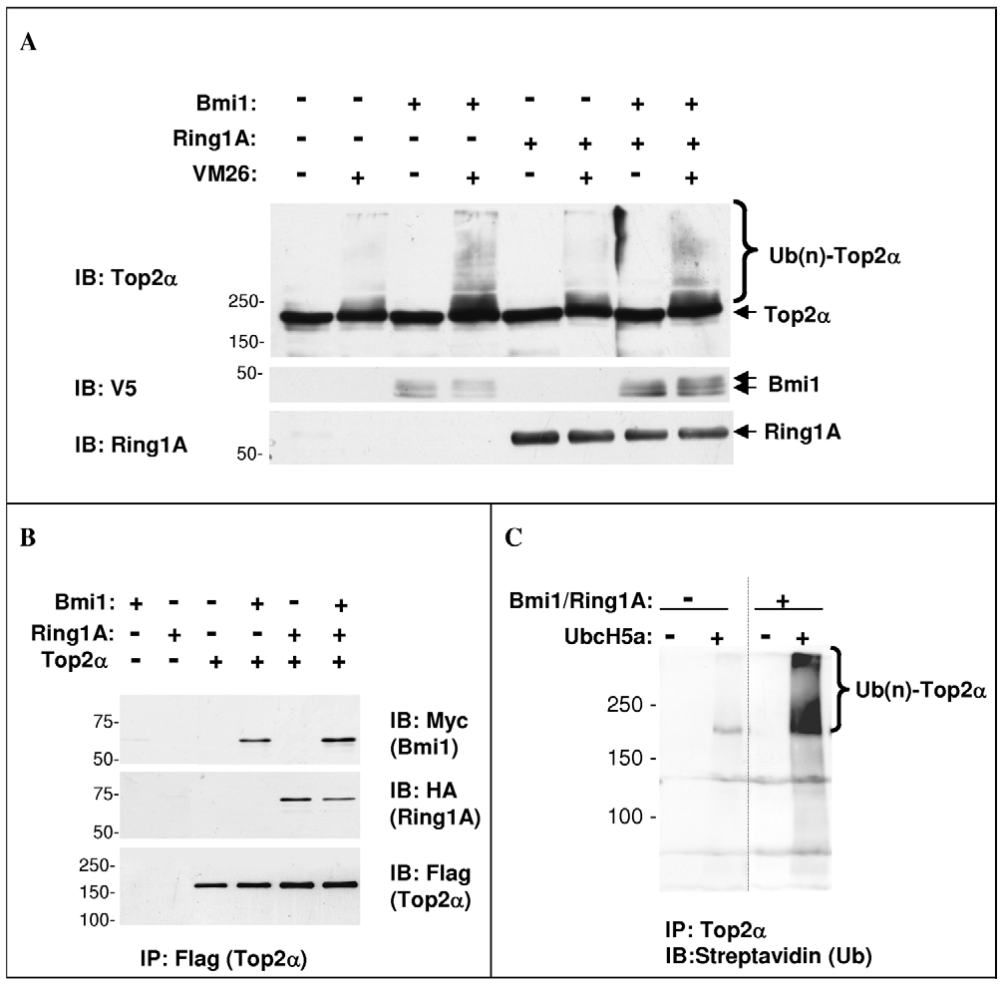
Bmi1 and Ring1A bind and ubiquitinate Top2α. A. HeLa cells were transfected with Ring1A and V5-tagged Bmi1 as indicated, and treated with 50 µM MG132 for 30 minutes with or without 100 µM VM26. The cells were extracted by hot lysis followed by sonication. Proteins were separated by 6.5% SDS-PAGE and Top2α was detected by Western-blot. The levels of transfected Bmi1 and Ring1A in the total extracts were detected using the indicated antibodies. B. Recombinant GST-myc-Bmi1 and GST-HA-Ring1A were incubated with or without recombinant FLAG-Top2α. At the end of the incubation Top2α was immuno-precipitated with anti-Flag conjugated beads and the bound proteins were detected by Western-blot as indicated in the figure. C. Flag-tagged Top2α was transfected into HeLa cells. Cell extracts were immuno-precipitated with anti-Flag conjugated beads. The immuno-precipitate was used as a substrate in an ubiquitination reaction, using recombinant Bmi1/Ring1A, UbcH5a and biotin-tagged ubiquitin. At the end of the incubation the beads were washed, the bound proteins were separated on 6.5% SDS-PAGE and blotted with streptavidin-tagged HRP.

To determine the effect of Bmi1 and Ring1A on Top2α ubiquitination in cells we transfected HeLa cells with Bmi1 and Ring1A separately or together. Cells were then treated with VM26 to induce Top2α ubiquitination and MG132 to prevent proteasomal degradation, and allow the accumulation of ubiquitinated Top2α species (Fig. 7A). We show that overexpression of Bmi1 is sufficient to increase the ubiquitination of Top2α, while Ring1A overexpression does not contribute additional ubiquitination. This suggests that Bmi1 is the limiting factor in Top2α ubiquitination in HeLa cells and is in agreement with various reports showing that Bmi1 is a key factor in the PRC1.

Next, to establish whether Bmi1 and Ring1A are capable of binding Top2α directly, we tested the binding of recombinant Bmi1 and Ring1A to recombinant Top2α. Binding reactions were set up with varying protein combinations. Top2α was immunoprecipitated with anti-Flag beads and attached proteins were detected by immuno-blot (Fig. 7B). Both Bmi1 and Ring1A are associated with Top2α in this cell-free binding assay, either when each one was added separately or when combined together. To test if Top2α is a direct ubiquitination target of Ring1A-Bmi1, we used Flag-tagged Top2α immunopurified from transfected HeLa cells as a substrate in a cell-free ubiquitination assay containing biotin-tagged ubiquitin, UbcH5a and Bmi1/Ring1A. At the end of the reaction Top2α bound to anti-Flag was washed extensively and resolved by SDS-PAGE and ubiquitinated forms were detected with Streptavidin-HRP (Fig. 7C). The results show that Top2α is efficiently ubiquitinated by the Bmi1/Ring1A E3 complex in a cell-free assay. These results together with the stabilization effect of Bmi1 and Ring1A siRNAs on drug-induced Top2α degradation, suggest that the Bmi1/Ring1A complex may function as a Top2α ubiquitin ligase.

### Identification of Bmi1/Ring1A Inhibitors

After identifying Bmi1/Ring1A, as an E3 ligase involved in drug-induced Top2α degradation, we initiated a campaign for the identification of small-molecule inhibitors of its ubiquitination activity. The assumption is that such inhibitors will diminish proteasomal degradation of Top2α thus increasing the potency of Top2 directed drugs. For this purpose, we modified the HTRF^®^-based Bmi1/Ring1A ubiquitination assay into high throughput format. Using this assay we screened a library of 56,000 diverse small-molecule compounds and identified several chemical structural families that inhibit Bmi1/Ring1A ubiquitination activity. Initial hits were tested using several cell-free filtering assays, designed to eliminate assay artifacts and inhibitors of other components in the reaction. Specifically we set up assays for measuring E1 dependent ubiquitin activation and E2 dependent ubiquitin conjugation. Bmi1/Ring1A inhibition was also measured using a gel-based method similar to that shown in figure 6B (data not shown).

**Figure 8:**
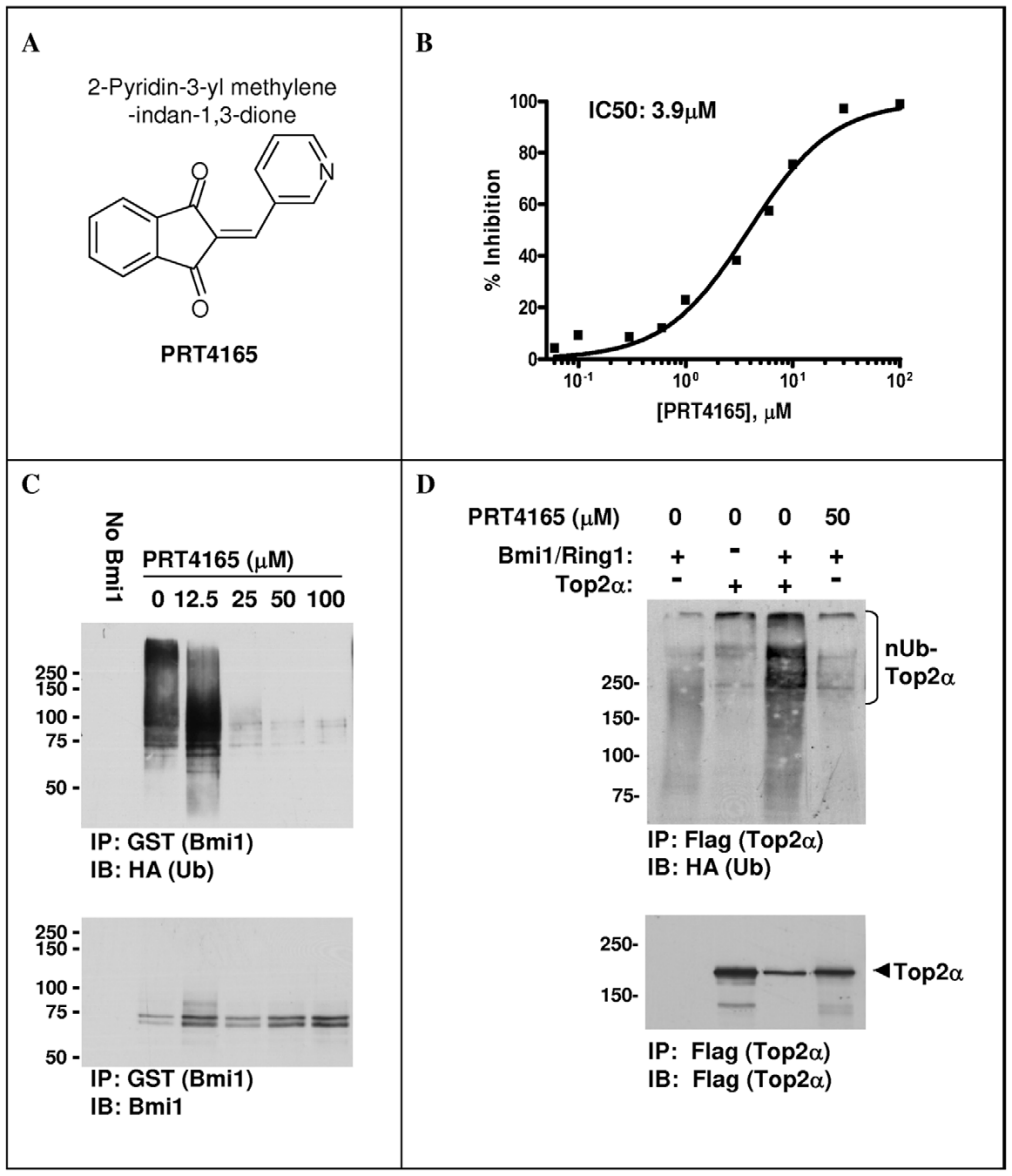
Compound PRT4165 inhibits both Bmi1/Ring1A self-ubiquitination and Top2α ubiquitination *in-vitro*. A. Chemical structure of PRT4165. B. Inhibition of Bmi1/Ring1A self-ubiquitination as detected by HTRF^®^ assay and determination of IC50 value. C. Inhibition of Bmi1-Ring1A self-ubiquitination by PRT4165 as detected by a Western-blot method. D. Inhibition of Bmi1/Ring1A-induced ubiquitination of immunopurified FLAG-Top2α. HeLa cells were transfected with FLAG-Top2α or empty vector. Twenty-four hours post transfection Top2α was immunopurified on anti-FLAG beads and used as a substrate for ubiquitination with recombinant Bmi1/Ring1A.

Following elimination of false positive hits, four different chemical scaffolds were identified. One of them is the Indan-1,3-dione family. Four compounds of this family were identified in the screen. Here we describe the results with one of these compounds, PRT4165 (Fig. 8A), that has an IC50 of 3.9 µM in the cell-free HTRF^®^ assay (Fig. 8B). In order to verify that the activity of PRT4165 is not an HTRF artifact, its activity was also tested by a Western-blot based ubiquitination assay (Fig. 8C), an assay that uses a completely different readout. The results of the gel-based assay demonstrate that the inhibition by PRT4165 is not dependent on the detection system. At 12.5 µM PRT4165 shortening of the ubiquitin chains is observed and at 25 µM ubiquitination is nearly completely eliminated. Next we tested if the inhibition of Bmi1/Ring1A ubiquitination activity is translated into inhibition of Bmi1/Ring1A dependent Top2α ubiquitination, in a cell-free system (Fig. 8D). We show that indeed PRT4165 also inhibits Bmi1/Ring1A mediated ubiquitination of Top2α.

### Cellular Activity of the Bmi1-Ring1A Inhibitor PRT4165

**Figure 9:**
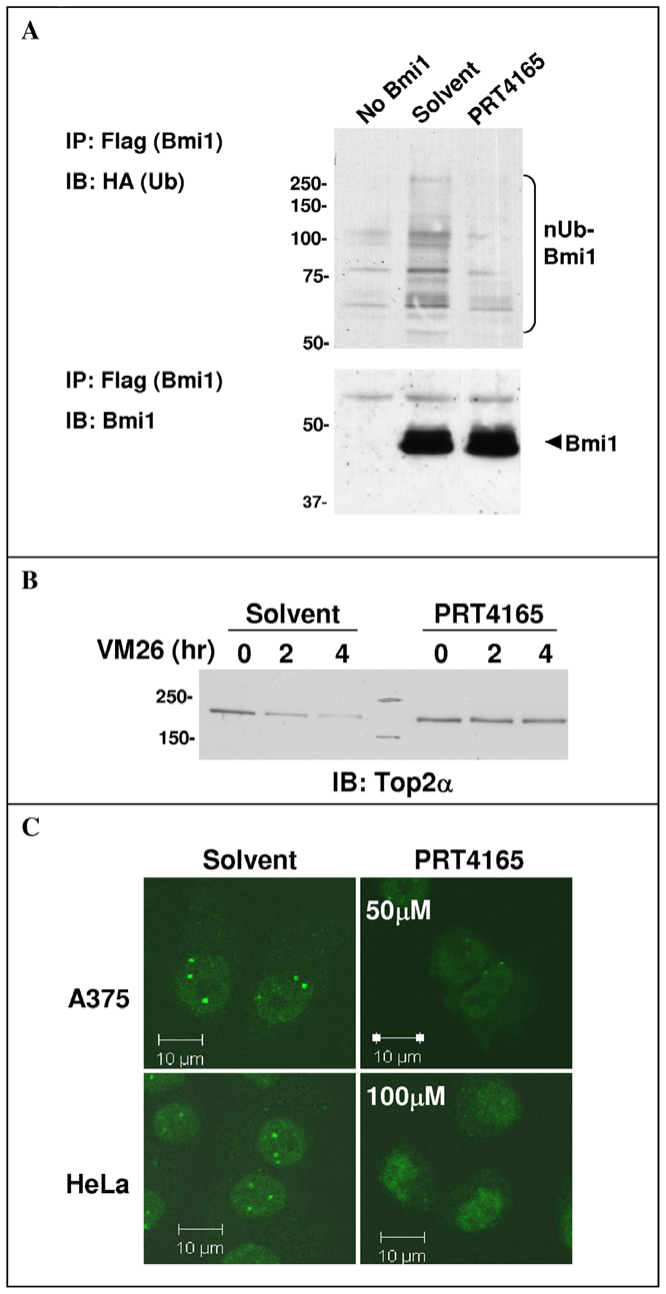
*In-vivo* activity of PRT4165. A. Inhibition of Bmi1 ubiquitination. HeLa cells were transfected with Bmi1-FLAG, Ring1A and HA-ubiquitin. Twenty-four hours post transfection the cells were treated with either solvent (0.5% DMSO, 0.5% PEG400) or 50 µM PRT4165 for 5 hours. Bmi1-FLAG was immunoprecipitated from cell lysates, and conjugated ubiquitin was detected by Western-blot with anti-HA antibody. B. Prevention of Top2α drug induced degradation. HeLa cells were treated with 100 µM and with either 50 µM PRT4165 or solvent for the indicated time. Top2α protein was recovered from the DNA by alkaline lysis and S7 nuclease treatment and its level was determined by Western-blot with Top2α antibody. C. Disruption of Bmi1 nuclear localization. Cellular localization of Bmi1 was determined by immunoflouresence using Bmi1 directed antibodies in HeLa and A375 cells, treated for 3 hours with the indicated concentrations of PRT4165 or solvent.

In order to test if PRT4165 inhibits Bmi1/Ring1A activity also in the context of a whole cell, we tested its effect on Bmi1/Ring1A self-ubiquitination activity in HeLa cells. The assay measures Ring1A dependent Bmi1 ubiquitination with exogenously expressed proteins. When Bmi1, Ring1A and HA-tagged ubiquitin are co-transfected into HeLa cells, conjugation of HA-ubiquitin to immunoprecipitated Bmi1 is observed (Fig. 9A). Treating cells for 5 h with 50 µM PRT4165 inhibits this ubiquitination. We assumed that PRT4165 inhibition of Bmi/Ring1A will not be limited to self-ubiquitination but will also inhibit VM26-induced Top2α ubiquitination and thus its proteasomal degradation. Toward that aim, HeLa cells were treated with either solvent or 50 µM PRT4165 combined with VM26 for the indicated periods (Fig. 9B). The total level of Top2α in the cells was determined after nuclease treatment. After 4 h of VM26 treatment, Top2α is mostly degraded, whereas 50 µM PRT4165 completely inhibits this VM26-induced degradation.

Bmi1 is reported to appear partly in diffuse nuclear staining and partly as nuclear speckles indentified as polycomb-group (PcG) bodies [25]. The localization of Bmi1 to the PcG bodies is dynamic and it was suggested that post-translational regulation such as phosphorylation or ubiquitination may be involved [25]. This led us to test whether PRT4165 affects Bmi1 localization in cells. Staining of cells with Bmi1 antibodies, results in a typical punctate appearance of endogenous Bmi1 (Figure 9C). We found that a 3 hour treatment with 50 µM PRT4165 in A375 cells or 100 µM in HeLa cells, leads to disappearance of the speckled staining in the nuclei and appearance of Bmi1 also in the cytoplasm (Figure 9C).

### PRT4165 Synergistically Increases Potency of Top2 Drugs

**Figure 10:**
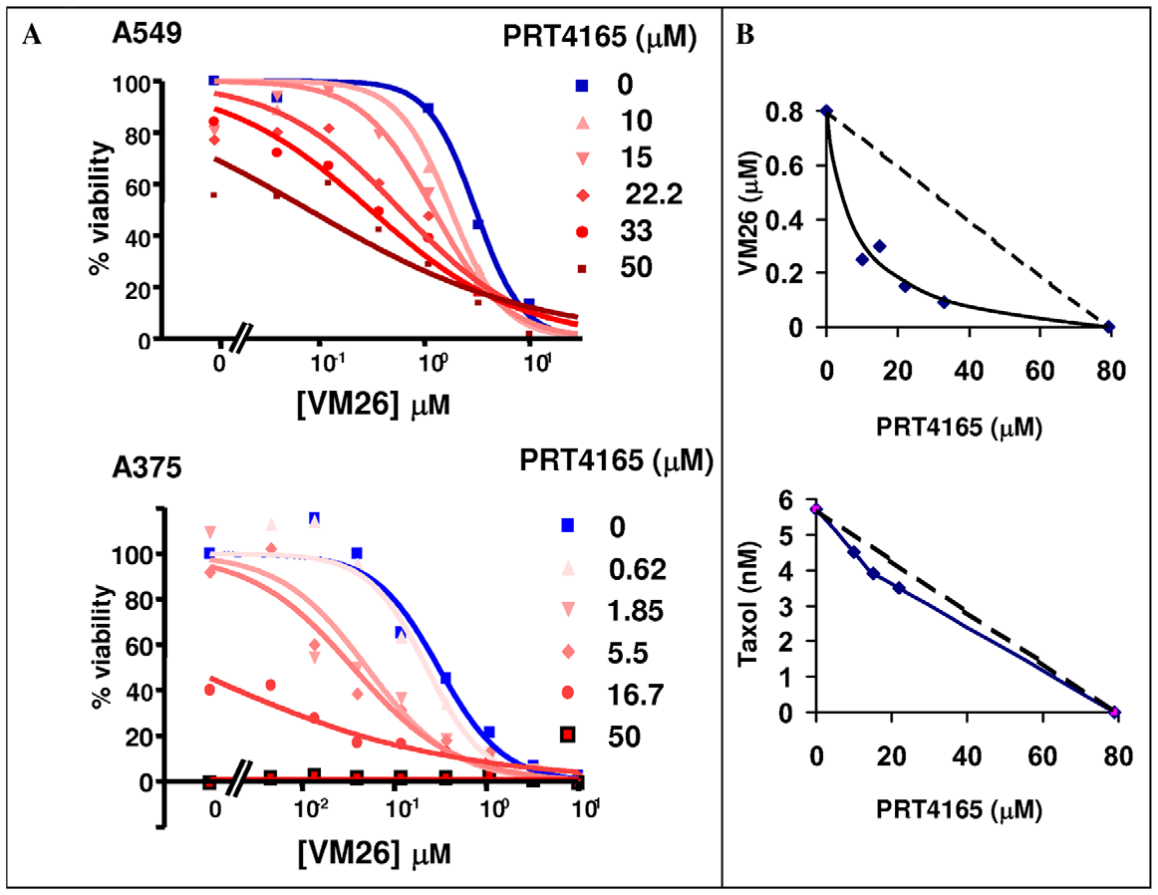
The cytotoxicity of VM26 is increased by PRT4165 in a synergistic manner. A. Dose dependent VM26 sensitivity of A549 lung carcinoma cells (top) and A375 malignant melanoma cells (bottom) in the presence of increasing amounts of PRT4165. The cells were treated with the drugs for twenty-four hours and then grown in medium without drugs for additional forty-eight hours before their viability was determined using WST1 reagent. B. Isobolgram analysis of combined drug sensitivity in A549 cells, using PRT4165 with either VM26 (top) or Taxol (bottom).

Next we set to test whether prevention of drug-induced Top2α degradation by PRT4165 is also translated into increased Top2 drug efficacy in these cells. Towards that end, we determined the effect of PRT4165 on the LD50 of VM26 in A549 lung cancer and A375 melanoma cells (Fig. 10A). Increasing concentrations of PRT4165 systematically improves the efficacy of VM26 in a dose-dependent manner. In A549 lung cancer cells (top panel) the LD50 of VM26 is reduced ten-fold from 3.1 µM when treated alone to 0.3 µM when VM26 is combined with 33 µM of PRT4165. In A375 melanoma cells (bottom panel) PRT4165 is even more effective, a similar ten-fold increase in VM26 efficacy is observed at a concentration of 5.5 µM PRT4165. Isobolgram analysis (Fig. 10B) demonstrates that the combined toxicity of PRT4165 and VM26 is synergistic (top panel) whereas the combined effect of PRT4165 with another commonly used cancer drug such as Taxol, that does not target topoisomerases, is additive (Fig. 10B, bottom panel). The synergistic effect PRT4165 is not limited to VM26 alone, but is observed also with other Top2 drugs such as doxorubicin (data not shown).

## Discussion

Here we describe, first the identification of Bmi1/Ring1A as a functional ubiquitin-ligase complex involved in Top2α degradation induced by either Top2 drugs or low glucose, and then the discovery of a small molecular weight inhibitor for this ligase. We show that Top2α is an ubiquitination target of Bmi1/Ring1A in a cell free system and that Bmi1 overexpression increases drug-induced Top2α ubiquitination in cells. Recently it was reported that the polycomb complex remains bound to DNA during DNA replication *in vitro* [26]. This localizes the polycomb complex at the right place and time to be a direct E3 ligase of Top2α when it is within the Top-DNA cleavage complex. However, whether or not Top2α is indeed a direct ubiquitination target of Bmi1/Ring1A also in cells still has to be shown by additional studies. Two other E3 ubiquitin ligases, Mdm2 and Brca1, were implicated in the ubiquitination and degradation of Top2α. Mdm2 binds to Top2α and mediates its ubiquitination and subsequent degradation following etoposide treatment [27]. Cells harboring a single nucleotide polymorphism that increase the expression of Mdm2 were found to be about ten-fold more resistance to Top2 poisons whereas silencing of Mdm2 increased sensitivity, although to a lesser extent. Several pieces of evidence connect Brca1 to Top2α ubiquitination. It was shown that Brca1 binds and ubiquitinates Top2α and this ubiquitination increases the DNA decatenation activity of Top2α [28]. In another report it was suggested that retinoblastoma protein (pRb) facilitates processing and repair of Top2-cleavable complexes by recruiting Brca1 to Top2α at the damaged site [29]. Oxidative stress also leads to Brca1 and pRb-dependent ubiquitination and subsequent degradation of Top2α in some cell lines [30]. The last two reports suggest that pRb serves as an adaptor protein to recruit Brca1 (and other proteins) involved in Top2α ubiquitination. Interestingly, pRb is also localized in the PcG bodies and interacts with Ring1A [31]. The exact roles filled by Mdm2, Brca1 and Bmi1/Ring1A, in the ubiquitination and degradation of Top2α is yet to be determined.

Self-ubiquitination is a mechanism that serves some E3-ligases as a mean to control their own level. Inhibiting Bmi1/Ring1A ubiquitination activity may raise the concern that this may lead to increased cellular levels of these oncogenic proteins. Prior to initiating the screen for inhibitors, we have noticed that co-expression of Bmi1 and Ring1A results in higher expression levels of both proteins (unpublished results). These results imply that the self-ubiquitination activity of Bmi1/Ring1A is not serving as a degradation signal, similar to the reported results for Bmi1 and Ring1B [24]. In agreement with this, it turns out, that the identified inhibitor PRT4165 does not increase the observed cellular levels of either Bmi1 or Ring1A (data not shown). However, application of PRT4165 does modify the cellular localization of Bmi1, from discrete PcG bodies within the nucleolus to disperse nuclear and even cytoplasmic localization. How the inhibitor leads to miss-localization of Bmi1 and whether it affects the oncogenic properties of Bmi1 remains to be explored.

Previous publications have demonstrated ubiquitination activity of Ring1A [19], [32] however this activity was very weak and the main ubiquitination activity of the PRC1 complex was attributed to Ring1B. Here we demonstrated a robust ubiquitination activity of Ring1A when it is in a complex with Bmi1. It appears that the contribution of Bmi1 does not depend on the integrity of its E2 binding site. An activation role of Bmi1 was also demonstrated in the case of Ring1B-dependent H2A ubiquitination [17], [24], [32]. Bmi1 has a key role in the function of the PRC1 complex as was reflected by its overexpression in many different tumors. In agreement, we show that Bmi1 overexpression, but not Ring1A overexpression, increases drug-induced Top2α ubiquitination. Taken together it may suggest that a key role of Bmi1 in the PRC1 complex is to activate the ubiquitination activity of PRC1 in a Ring1A or a Ring1B-dependent manner. In addition it may suggest a modular mode of action, where the level of activity is determined by the expression of Bmi1, and the relative levels of the other RING-domain subunits influence the target selectivity.

Bmi1 is a key component of PRC1 regulating chromatin remodeling and gene expression pathways essential for self-renewal of stem cells and cancer stem cells [33], [34], [35], [36]. Bmi1 was first identified as an oncogene inducing B and T cell leukemias [37], [38] and later it has been repeatedly shown to be highly overexpressed in various cancer cell-lines and tumors (reviewed in [39], [40]). Moreover, silencing of Bmi1 by siRNA leads to the death of cancer cells specifically [12], suggesting that its activity is also essential for viability during the malignant stage. Ring1A has been also shown to have tumorogenic properties as its overexpression leads to anchorage-independent growth and tumor induction in athymic mice [18], however its expression does not seem to be upregulated in tumors [41]. In contrast, Ring1B expression is increased in various types of tumors [41]. The PRC1 complex with its core activity of an ubiquitin ligase, cooperates with the PRC2 complex, with a core activity of histone methyltransferase [42] and association with DNA methyltransferases (DNMTs) activity [43]. A key component in the PRC2 complex is EZH2 histone methylase and similar to Bmi1, it is also elevated in various cancers [42]. PRC1 and PRC2 act together to epigenetically silence target genes [44], many of them with pro-differentiation and anti-proliferative function [45]. The exact role of the different polycomb complexes, the relation between the methylation and ubiquitination and the sequence of events are under extensive research by many labs. However, the picture that emerges is that these complexes and their activities play a crucial role in the initiation and maintenance of cancer and stem cell phenotype of cancer cells. Hence, the ubiquitination and methylation activities of the polycomb complexes appear as promising therapeutic targets for cancer therapy and moreover, for targeting cancer-stem-cell self-renewal. Therefore, the effect of PRT4165 on the PRC1-repressed genes and the self-renewal capabilities of cancer stem cells would be highly relevant to its therapeutic potential.

Inhibiting Bmi1/Ring1A is expected to increase the persistence of Top2α-DNA cleavage complex, by preventing its proteasomal degradation, leading to increased potency of Top2 drugs. Such a combination therapy can be beneficial, as increasing the potency of such a drug by this mechanism, can either achieve better eradication of cancer cells using the same amount of drug, or allow the use of lower doses. This can reduce some of the side effects associated with these cytotoxic drugs. As Top2α is expressed only in proliferating cells [46] and is overexpressed in many cancer cells, a combination therapy of an anthracycline and a Bmi1/Ring1A inhibitor, is expected to achieve similar specific killing of cancer cells through increased poisoning of Top2α, with reduced unwanted side effects.

The ubiquitin system of protein modification is a crucial mechanism involved in almost every aspect of cellular processes. The evidences for the involvement of the ubiquitin system in human diseases are rapidly accumulating reflecting the central role of the system in cellular function. However, the concept that E3 ubiquitin ligases are druggable targets is still to be demonstrated. The complexity of the assays and the lack of classic enzymatic activity make the task even more challenging. Bmi1 represents one of the attractive potential targets in the ubiquitin system, either for a stand-alone therapy or as we show here, for combination therapy with Top2 poisons.

## Supporting Information

Table S1 siRNA used in the study
(0.02 MB DOC)

Figure S1 Dependence of Bmi1-Ring1A self-ubiquitination on concentration of various E2 conjugating enzymes. Ubiquitination of co-expressed GST-Bmi1 and Ring1A, with varying amounts of different E2 enzymes detected by HTRF®.
(2.75 MB TIF)
