# Supplementary material for: The E3 Ubiquitin-Ligase Bmi1/Ring1A Controls the Proteasomal Degradation of Top2α Cleavage Complex – A Potentially New Drug Target
Source: PLoS One. 2009 Dec 1;4(12):e8104. doi: 10.1371/journal.pone.0008104 (PMC2779455; doi:10.1371/journal.pone.0008104)
Supplement: Table S1 — siRNA used in the study (0.02 MB DOC) [file pone.0008104.s001.doc]

Table S1: siRNA used in the study

| Code | Gene | Accession | Position (from ATG) | Sense strand | Anti-sense strand |
| --- | --- | --- | --- | --- | --- |
| X63 | BMI-1 | NM_005180.5 | 952 | AUGGGUCAUCAGCAACUUCUUCUdGdG | CCAGAAGAAGUUGCUGAUGACCCAUUU |
| X164 | BMI-1 | NM_005180.5 | 163 | UUUGUGAUGUCCAAGUUCACAAGdAdC | GUCUUGUGAACUUGGACAUCACAAAUU |
| X165 | BMI-1 | NM_005180.5 | 218 | AACUCUCCAAGAUAUUGUAUACAdAdA | UUUGUAUACAAUAUCUUGGAGAGUUUU |
| X154 | RING1A | NM_002931.3 | 212 | CUGCAUUGUCACAGCCCUACGGAdGdC | GCUCCGUAGGGCUGUGACAAUGCAGUU |
| X155 | RING1A | NM_002931.3 | 325 | AGAUCUAUCCUAGCCGGGAGGAAdTdA | UAUUCCUCCCGGCUAGGAUAGAUCUUU |
| X96 | RING1B | NM_007212.3 | 1001 | GCACAAAUGAGCCUUUAAAAACCdAdA | UUGGUUUUUAAAGGCUCAUUUGUGCUU |
