# Supplementary figures and images for: The E3 Ubiquitin-Ligase Bmi1/Ring1A Controls the Proteasomal Degradation of Top2α Cleavage Complex – A Potentially New Drug Target
Source: PLoS One. 2009 Dec 1;4(12):e8104. doi: 10.1371/journal.pone.0008104 (PMC2779455; doi:10.1371/journal.pone.0008104)

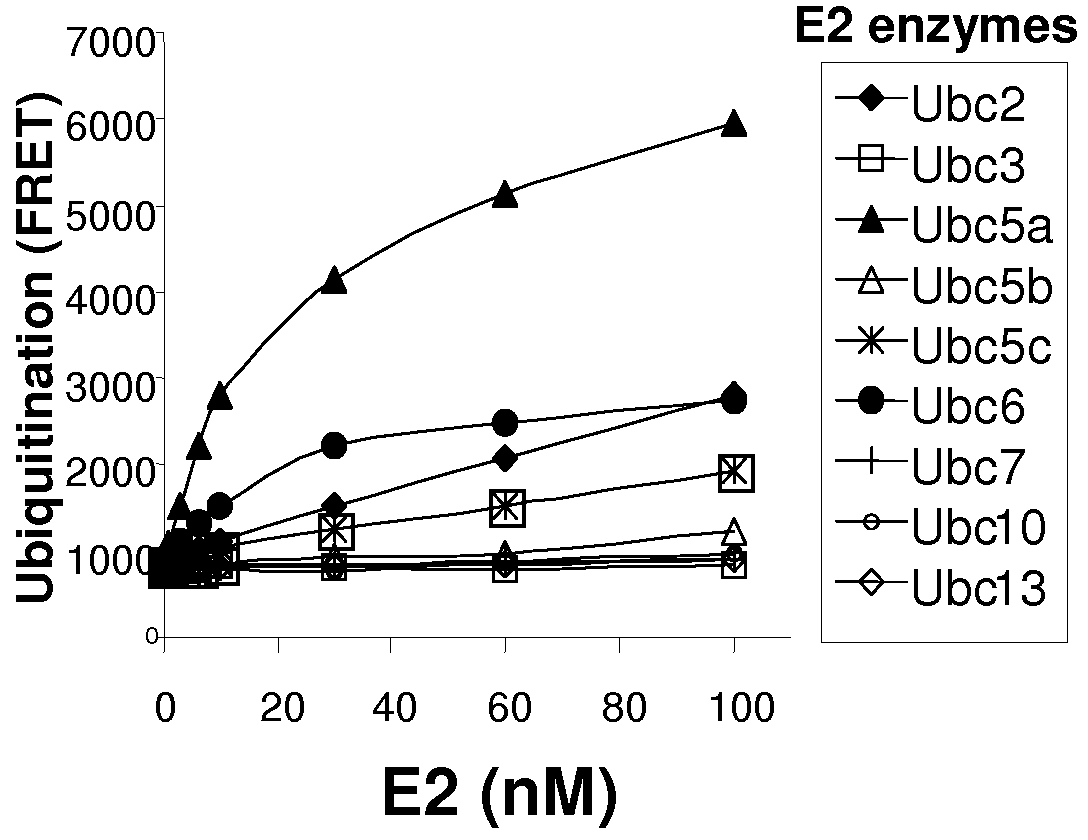

Supplement: Figure S1 — Dependence of Bmi1-Ring1A self-ubiquitination on concentration of various E2 conjugating enzymes. Ubiquitination of co-expressed GST-Bmi1 and Ring1A, with varying amounts of different E2 enzymes detected by HTRF®. (2.75 MB TIF) [file pone.0008104.s002.tif]
